# A short course of high-resistance, low-volume breathing exercise extends respiratory endurance and blunts cardiovascular responsiveness to constant load respiratory testing in healthy young adults

**DOI:** 10.1016/j.resp.2022.103974

**Published:** 2022-09-27

**Authors:** Claire M. DeLucia, Dallin Tavoian, Dean R. Debonis, E. Wyatt Snell, Sarah M. Schwyhart, E. Fiona Bailey

**Affiliations:** Department of Physiology, University of Arizona College of Medicine, Tucson, AZ 85721-0093, USA

**Keywords:** Respiratory training, Pressure time, Blood pressure

## Abstract

Our objective was to evaluate the effects of 6-weeks high-resistance, low-volume inspiratory muscle strength training (IMST) on respiratory endurance, blood pressure (BP) and heart rate (HR) responsiveness to high respiratory workloads. Ten healthy young adults completed **two** constant-load resistive breathing tests to exhaustion (T_*lim*_) (target pressure =65 % maximal inspiratory pressure [PI_max_]; duty cycle = 0.7; breathing frequency matched to eupnea) separated by 6-weeks high-resistance (75 % maximal inspiratory pressure, PI_max_), low-volume (30 inspiratory efforts/day, 5 days/week) IMST. Throughout resistive breathing trials we measured beat-to-beat changes in BP and HR, mouth pressure, inspiratory muscle work and perceived exertion. POST resistive breathing tests revealed significant gains in endurance (PRE: 362.0 ± 46.6 s vs. POST: 663.8 ± 110.3 s, p = 0.003) and increases in respiratory muscle work (PRE: −9445 ± 1562 mmHg.s vs. POST: −16648 ± 3761 mmHg.s, p = 0.069). Conversely, systolic and diastolic BP responses, HR and ratings of perceived exertion all declined. Consistent with previous observations, 6 weeks high resistance, low volume IMST lowered casual *resting* SBP (p = 0.002), DBP (p = 0.007) and mean arterial pressure (p = 0.001) and improved static inspiratory pressure. High resistance, low volume inspiratory muscle strength training extends respiratory endurance and attenuates BP responsiveness in healthy, recreationally-active young adults. The outcomes have implications for improved athletic performance and for attaining and/or maintaining cardiorespiratory fitness.

## 1. Introduction

Previous studies have implemented high-resistance (75 %PI_max_), low-volume (5 mins/day, 5 days/week) inspiratory muscle strength training (IMST) in healthy young adults and reported reductions in systolic blood pressure (SBP; −4 to 6 mmHg) (DeLucia et al., 2018; Vranish and Bailey, 2015) and systemic vascular resistance (−3.5 mmHg.min/l^−1^) (DeLucia et al., 2018), accompanied by enhanced inspiratory muscle strength (average ~25–35 mmHg gain in PI_max_) (DeLucia et al., 2018; Vranish and Bailey, 2015) and acute suppression of muscle sympathetic nerve activity (averag ~40 % decline) (DeLucia et al., 2021).

The purpose of the current study was to assess the effects of high-resistance low-volume IMST on inspiratory muscle strength, respiratory endurance (T_*lim*_) and cardiovascular responsiveness to high respiratory muscle workloads. In accordance with protocols designed to elicit inspiratory muscle fatigue (Bellemare and Grassino, 1982a and b; Sheel et al., 2001), we used constant-load resistive breathing tests to evaluate the effects of 6-weeks high-resistance, low-volume IMST on; a) T_*lim*_, b) inspiratory muscle work (cumulative pressure time product [PTP]), and c) cardiovascular responsiveness (i.e., heart rate [HR] and blood pressure [BP]) to exhaustive respiratory muscle exercise. We also evaluated casual resting BP and static gains in respiratory muscle strength (i.e., PI_max_).

## 2. Methods

Twelve casual exercisers who were non-smokers, normotensive and free from cardiovascular disease, were recruited from the student population at the University of Arizona. Ten subjects (3 women, 7 men; 21.1 (± 2.5) years; BMI 21.8 (± 2.7)) completed the study (Table 1). Two subjects discontinued due to the COVID pandemic and were unable to resume study participation. Endurance task data from one male subject were excluded from consideration due to significant pre-post differences in PTP/breath. Experimental procedures were approved by the University of Arizona Human Subjects Protection Program and in accordance with the *Declaration of Helsinki,* all subjects provided their written informed consent.

Resting BP and PI_max_ were assessed prior to each resistive breathing test and, at the end of each training week. BP was assessed after 5 min seated rest with three measures averaged to obtain SBP and diastolic BP (DBP) and mean arterial pressure (Pickering et al., 2005). Three maximal inspiratory efforts against near-infinite resistance were averaged to determine PI_max_ in accordance with standardized procedures (American Thoracic Society/European Respiratory, 2002).

In each resistive breathing test (RBT) to exhaustion, subjects breathed continuously via a constant-load circuit comprising a flanged mouthpiece attached to a non-rebreathing valve fitted with a flow limitation end cap. For RBTs, target inspiratory pressures were set to 65 % PI_max_ with an extended inspiratory phase duration equivalent to 0.7 T_I_/T_TOT_ (where T_I_/T_TOT_ is the duty cycle) (Bellemare and Grassino, 1982a and b; Sheel et al., 2001). Breathing rate was set at the baseline eupneic breathing frequency.

Throughout each test, subjects viewed a real-time display of mouth pressure and target inspiratory pressure and ramp audio tones cued breath phase durations. *T*_*lim*_ was defined as the time point at which the subject no longer was able to initiate a breath *or* when inspiratory muscle work (i.e., PTP/breath) declined by ≥ 10 % (relative to average PTP/breath in minute 1.0 of the test) for three consecutive breaths. Signals were sampled at 500 Hz, digitized, and stored using a Cambridge Electronic Design 1401 interface and software (Cambridge Electronic Design, Cambridge, UK). Beat-to-beat changes in BP were sampled via an automated finger cuff pressure transducer (ccNexfin; Bmeye, Amsterdam, The Netherlands) on the non-dominant hand. Continuous lead-II ECG was sampled using surface electrodes (Kendall 133 foam electrodes; Covidien, Mansfield, MA) and recorded online (LabChart 8.0, ADInstruments. Colorado Springs, CO). Participants registered ratings of perceived exertion (RPE) at 30-second intervals via laser pointer directed to a 15-point Borg scale.

All subjects completed 6 weeks high resistance, low volume IMST in the laboratory on a two-way non-rebreathing valve (2600 series, Hans Rudolph, Shawnee, KS). A flow limitation end cap on the inhalation port provided a constant, near-maximal inspiratory resistance, and restricted airflow to a pin-hole leak. A tube attached to the device and coupled to a pressure transducer (Omegadyne Inc., Sunbury OH) detected airway opening pressure. Subjects first exhaled to residual lung volume and then inhaled against a constant resistance to the target training pressure (75 % PI_max_) displayed on a monitor. Subjects were guided to achieve that target and to hold the target pressure for 1–2 s and then exhale to residual volume. There was no resistance to expiration. PI_max_ was reassessed at the end of each training week, and target resistance was adjusted accordingly. Importantly, training pressures (i.e., 75 % of PI_max_) and resistive breathing test pressures (i.e., 65 % PI_max_) differed.

### 2.1. Data analysis

PTP/breath (mmHg.s) was summed to obtain cumulative PTP for pre and post-resistive breathing tests (American Thoracic Society/European Respiratory, 2002). Beat-to-beat measures of SBP, DBP and HR were averaged during the 5 min of baseline recordings prior to each resistive breathing test, and BPs and HRs expressed relative to that average (% baseline). Data for PTP, BP, HR, and RPE were normalized on the basis of the total breath number for pre and post resistive breathing tests and expressed as an average for each test quartile (i.e., 0–25, 25–50, 50–75 and 75–100 %test).

### 2.2. Statistical analysis

Analyses were completed in SPSS V 26.0. Intervention-related changes in primary outcome measures (SBP, DBP, MAP, HR, cumulative PTP, PI_max_, and *T*_*lim*_) were assessed with paired t-tests at α = 0.05, and changes reported as average ± SEM. Corrected Hedges’ g effect sizes for small samples were generated to quantify the effect(s) of the intervention on the primary outcome measures. Effect sizes were defined as: very small/no effect (<0.20), small (0.20–0.49), moderate (0.5–0.79), large (0.8–1.19), and very large (≥1.20) (Sawilowsky, 2009).

## 3. Results

Inspiratory loads in pre/post RBTs were equivalent and set at 65 % of pre-intervention PI_max_. Accordingly, subjects performed the same inspiratory muscle work per breath in pre- and post-resistive breathing tests (PRE: 130.5 ± 61.1 mmHg.s versus; POST: −134.7 ± 68.2 mmHg; p = 0.236; ES=0.06). Despite comparable PTP per breath, average *T*_*lim*_ was significantly longer post- vs pre-test (p = 0.003; ES=1.13). Average cumulative PTP also was markedly greater post than pre-testing however, the increase failed to attain statistical significance (p = 0.069; ES = 0.64) (Table 1).

**Fig. 1.**
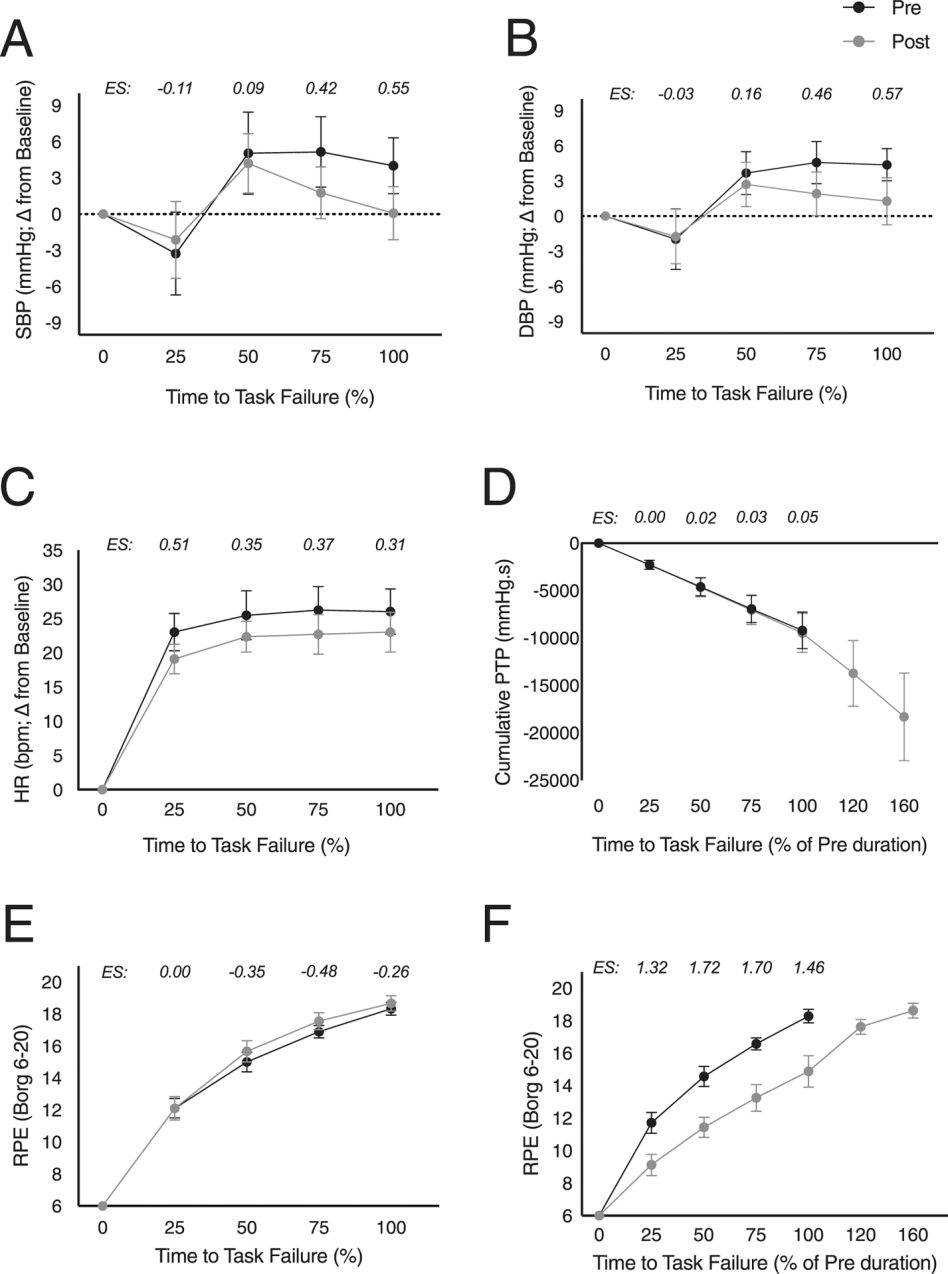
Change in systolic blood pressure (SBP; Panel A), diastolic blood pressure (DBP; Panel B), heart rate (HR; Panel C), cumulative inspiratory work (PTP; Panel D), ratings of perceived exertion (RPE; Panel E) and RPE expressed as a percentage of pre-intervention trial duration (Panel F) recorded during the RBT_PRE_-(black) and RBT_POST_-(grey). Values are plotted as a percentage of the pre- (black) or post- (gray) intervention endurance test time and expressed relative to baseline levels recorded prior to the start of the RBT. Values are mean ± SEM. Hedges’ G effect sizes (effect of intervention) are shown above plotted points.

Comparisons across quartiles between pre/post-tests (%trial) revealed lowered SBP post testing, with very small effects at 25 % and 50 %, a small effect at 75 % and a moderate effect at 100 % of the resistive breathing test (Fig. 1A). DBP similarly was lower post-test with very small effects at 25 % and 50 %, a small effect at 75 %, and a moderate effect size at 100 % of the test (Fig. 1B). Some attenuation of HR was evident post-test with a moderate effect size at the 25th quartile but otherwise small effects at 50 % and 100 % (Fig. 1C). There was no change in RPE as a function of test duration (Fig. 1E), however, when compared at the equivalent work output of the pre-test (%pre) RPE post-test were lower at all quartiles (Fig. 1F).

We also report significant training-related improvements in PI_max_ (*p* = 0.002; ES=1.85), and declines in resting SBP (*p* = 0.002; ES=0.57), DBP (*p* = 0.007; ES=1.03) and MAP (*p* = 0.001; ES=0.92) (Table 1).

## 4. Discussion

Numerous studies have assessed the impact of respiratory muscle training on exercise performance in sport-specific contexts (e.g., rowing to exhaustion) (Illi et al., 2012; Karsten et al., 2018; Sales et al., 2016; Shei et al., 2018). However, the objective in this study was to define the effects of 6-weeks *high-resistance, low-volume* IMST on cardiovascular responsiveness to high respiratory workloads absent large (limb) muscle group activation. Like previous studies (Mickleborough et al., 2010; Welch et al., 2018), subjects breathed against a *constant-load* circuit (target pressure =65 %PI_max_; duty cycle = 0.7 T_i_/T_TOT_; breathing frequency matched to eupnea) to exhaustion. The results achieved with this higher resistance but more abbreviated protocol include the extension of T_*lim*_ by 83 %, attenuation of BP responsiveness to respiratory fatigue and attenuation of perceived exertion, improved inspiratory muscle strength and reductions in *resting* SBP and DBP 5.6 and 5.5 mmHg, respectively.

Increases in T_*lim*_ and cumulative PTP reflect a substantial improvement in respiratory endurance. Moreover, gains in respiratory work were paired with diminished cardiovascular responsiveness (i.e., SBP, DBP and HR; Fig. 1A–C). As posited previously (McConnell, 2012; Welch et al., 2018), such blunting may be the result of training-induced reductions in sympathetic outflow which has been shown acutely (DeLucia et al., 2021) and in the intermediate-term (Ramos-Barrera et al., 2020) in response to IMST.

Perceived exertion also contributes to athletic performance (Grant et al., 1999). Ratings of perceived exertion were equivalent pre- and post-test when expressed relative to the duration of the trial (Fig. 1E). However, expressed as a function of cumulative work output during pre-testing, exertion ratings were lower at equivalent work outputs during post-testing. That is, at comparable workloads, 6 weeks IMST induced reductions in perceived effort (Fig. 1F). Notably, the outcomes achieved with an abbreviated training regimen are consistent with results obtained with more traditional high-intensity aerobic exercise training (Gething et al., 2004; Hill et al., 1987) and IMST protocols (Romer et al., 2002; Volianitis et al., 2001).

As anticipated, we report significant training-related declines in *resting* SBP (DeLucia et al., 2018; Vranish and Bailey, 2015), as well as DBP, a *de novo* finding in healthy young adults. The 59 % increase in respiratory strength is greater than anticipated based on previous outcomes using the same training protocol (DeLucia et al., 2018; Ramos-Barrera et al., 2020; Vranish and Bailey, 2015, 2016) and exceeds results achieved with lower-resistance but higher-volume IMST protocols (Guenette et al., 2006; Kilding et al., 2010; Witt et al., 2007).

### 4.1. Practical applications

In addition to lowering resting blood pressure and improving inspiratory muscle strength, six weeks high-resistance, low-volume IMST extended the capacity for respiratory work and endurance in healthy, recreationally-active young adults. The outcomes have implications for athletic conditioning and for attaining and maintaining cardiorespiratory fitness.

## Figures and Tables

**Table 1:** Anthropometrics, cardiovascular and respiratory variables before and after 6 weeks of IMST.

	Pre Intervention	End Intervention	*p*-values
Anthropometries			
Sex (M/F)	7/3	–	–
Age (yrs)	21.1 (2.5)	–	–
Mass (kg)	68.3 (12.4)	–	–
BMI (kg/m^2^)	21.8 (2.7)	–	–
Cardiovascular			
SBP (mmHg)	119.0 ± 2.9	113.4 ± 3.3	**0.002**
DBP (mmHg)	75.0 ± 1.4	69.5 ± 2.0	**0.007**
MAP (mmHg)	89.7 ± 1.6	84.1 ± 2.2	**0.001**
HR (bpm)	70.5 ± 3.7	68.3 ± 3.4	0.530
Respiratory			
PImax (cmH_2_O)	−64.6 ± 5.1	−102.5 ± 7.6	**0.001**
t_lim_ (s)	362.0 ± 46.6^†^	663.8 ± 110.3^†^	**0.003**
PTP per Breath (mmHg.s)	−130.5 ± 20.4^†^	−134.7 ± 22.7^†^	0.236
Cumulative PTP (mmHg.s)	−10,585 ± 2200^†^	−16,809 ± 3756^†^	0.069

Anthropometric data are reported as mean (SD) while cardiovascular and respiratory variables are reported as mean ± SEM.

† Indicates n = 9.
